# Influence of Conductivity and Dielectric Constant of Water–Dioxane Mixtures on the Electrical Response of SiNW-Based FETs

**DOI:** 10.3390/s140202350

**Published:** 2014-01-29

**Authors:** Marleen Mescher, Aldo G.M. Brinkman, Duco Bosma, Johan H. Klootwijk, Ernst J.R. Sudhölter, Louis C.P.M. de Smet

**Affiliations:** 1 Chemical Engineering, Delft University of Technology, 2628 BL Delft, The Netherlands; E-Mails: mmescher@gmail.com (M.M.); a.g.m.brinkman@tudelft.nl (A.G.M.B.); d.bosma@tudelft.nl (D.B.); e.j.r.sudholter@tudelft.nl (E.J.R.S.); 2 Philips Research Laboratories, 5656 AE Eindhoven, The Netherlands; E-Mail: johan.klootwijk@philips.com; 3 Materials innovation institute M2i, 2628 CD Delft, The Netherlands

**Keywords:** silicon nanowire, field-effect transistor, liquid gate, back gate, conductivity

## Abstract

In this study, we report on the electrical response of top-down, p-type silicon nanowire field-effect transistors exposed to water and mixtures of water and dioxane. First, the capacitive coupling of the back gate and the liquid gate via an Ag/AgCl electrode were compared in water. It was found that for liquid gating smaller potentials are needed to obtain similar responses of the nanowire compared to back gating. In the case of back gating, the applied potential couples through the buried oxide layer, indicating that the associated capacitance dominates all other capacitances involved during this mode of operation. Next, the devices were exposed to mixtures of water and dioxane to study the effect of these mixtures on the device characteristics, including the threshold voltage (*V*_T_). The *V*_T_ dependency on the mixture composition was found to be related to the decreased dissociation of the surface silanol groups and the conductivity of the mixture used. This latter was confirmed by experiments with constant conductivity and varying water–dioxane mixtures.

## 1. Introduction

Silicon-based nanowire devices have been the subject of extensive research in the last decade. Most of the work focuses on different aspects of device fabrication and on (potential) sensor applications for the label-free detection of (bio)chemical species [1–4]. Studies on (bio)chemical sensing typically require (bio)chemical modification of the nanowire surface [5]. In addition, there is a large series of studies on the description of fundamental performance limits of nanowire-based devices [6,7], charge screening effects [8–10], improvement of the signal-to-noise ratio [11–13], the effect of surface modification on the nanowire electrical properties [14,15], and work on the incorporation of a reference electrode [16,17].

Soon after their introduction in 2001 [18], devices based on silicon nanowires (SiNWs) were applied in sensing experiments, addressing the pH sensitivity of silicon oxide-covered SiNWs as well as the detection of streptavidin binding on biotin-modified nanowires. The sensing mechanism was rationalized by considering the type of doping present in the SiNW and the changes in charge density at the sensor interface. The surface potential as a result of the surface charge density offsets the front and/or back gate potential and leads to a change of majority charge carriers in the SiNW. By far, most of the studied target compounds are charged and studied in an aqueous environment. Examples include not only protons and antibodies/antigens [18], but also deoxyribonucleic acid (DNA) [15,19], polyelectrolytes [20] and ions [21]. Recently it was shown that the response of so-called nanoISFET pH sensors can be described by analytical models [22], similar to those developed for describing the operation of ISFETs [23].

Since 2007 the responses of SiNW-based devices—which behave like field-effect transistors (FETs)—to uncharged target species in the gas or vapour phase have also been studied. This was first shown by Heath and his co-workers who studied the exposure of NO_2_, acetone and hexane in nitrogen (N_2_) to bare and silane-modified SiNWs [24]. Later, Engel *et al.* prepared aminopropyl-terminated SiNWs to detect trinitrotoluene (TNT) in N_2_ [25]. Over the past few years, Haick and co-workers reported an interesting series of research papers on the fundamentals and applications of functionalized SiNW-based FETs for the detection of polar (water, ethanol, 1-butanol, 1-hexanol, 1-octanol and 1-decanol) and nonpolar (*n*-hexane, *n* octane and *n*-decane) volatile organic compounds (VOCs) in oil-free air having 15% relative humidity [26–31]. This in-depth work shows that one of the sensing mechanisms involved is related to the changes in the surrounding dielectric medium due to the condensation of VOCs on the functionalized SiNW surface [29]. The surrounding dielectric effect is also believed to play a role in an interesting contribution of the Nokia Research Center in which etched SiNW-based devices were exposed to (neat) vapours of water, acetone, methanol, ethanol and 2-propanol in air [32].

In the present study we investigated the effect of exposure of the SiNWs to different solvents and the dielectric coupling in more detail. We used well-defined, top-down prepared, p-type SiNW-based devices. The SiNWs were covered with SiO_2_ and were not further modified. Their electrical response to binary, liquid mixtures of water and dioxane, having a range of dielectric constants (*ε*_r_ varies between 2 and 80), was studied. In contrast to the detection of vapours or gases described in the previous paragraph, the analysis of the FET responses in the liquid environment, allows one to apply liquid gating (*i.e.*, front gating) next to back gating. In this article we compared and discussed the influence of type of gating in aqueous solutions. Several papers have discussed the use of back gating and methods for liquid gating (e.g., on-chip Au and Pt electrodes, Ag/AgCl electrodes, extended off-chip gates [33–35]), but a direct comparison on the influence of the type of gating on the *I*_D_-*V*_GS_-characteristics has not been made before. Next, the devices were exposed to two different types of water–dioxane mixtures: as-prepared and mixtures with a constant electrical conductivity achieved by the controlled addition of a salt. The electrical characteristics of the devices when exposed to these conditions were investigated and discussed.

## 2. Experimental Section

**Figure 1.**
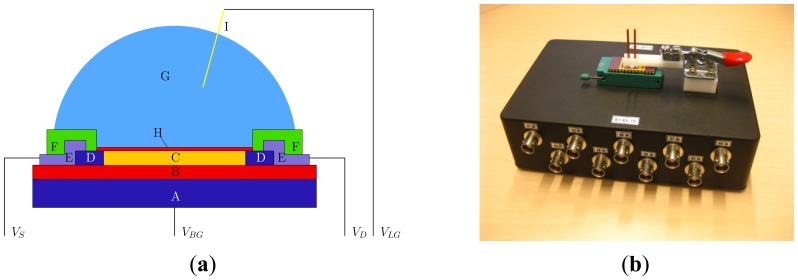
(**a**) Schematic representation of the experimental setup (not to scale). Atop of the high-doped (10^20^ cm^−3^) silicon back gate (A) and a 300 nm thick buried oxide layer (B), the low-doped (10^16^ cm^−3^) silicon nanowire is located (C). The ends of the nanowire consist of high-doped (10^20^ cm^−3^) silicon and form the source and drain contacts (D), which were contacted via aluminum contacts (E). The source, drain and back gate contacts were insulated using a 100 nm thick silicon nitride passivation layer (F), such that the nanowire and a certain area around it can be exposed to the solution of interest (G). Furthermore, the nanowire is covered with an 8 nm thick thermal silicon dioxide layer (H). An Ag/AgCl electrode (I) was placed at a fixed position in the solution; (**b**) Photograph of the box used for the electrical characterization. The cables and tubing are left out for clarity.

SiNW-FETs were produced as reported previously [36]. Briefly, the nanowires (p-doped at a concentration of 10^16^ cm^−3^ to assure semiconducting behaviour) are 3 μm in length, 300 nm in width and 40 nm in height and are covered with a silicon dioxide gate oxide with a thickness of 8 nm. The thickness of the buried oxide (BOX) layer is 300 nm. The devices were wire bonded and covered with a micro fluidic device. The setup is shown in Figure 1.

In order to investigate the difference between the use of a back gate (BG) and a liquid gate (LG), a Ag/AgCl electrode was inserted in the beaker containing the solution to which the nanowire was exposed. The solutions were sucked into the microfluidic cell with a syringe pump (Harvard Apparatus, Holliston, MA, USA) using Silastic^®^ Q7-4750 tubing (Dow Corning, Midland, MI, USA). The devices were exposed to the solutions in a random order. The target solution was sucked from the beaker into the microfluidic cell after which the flow was stopped and the measurements were performed under stagnant conditions at room temperature. The measurement was started immediately after exposure to the liquid. Each exposure continued for approximately 10 min using the liquid gate as described previously.

A standard Keithley 4200 semiconductor characterization system (Keithley Instruments BV, Gorinchem, The Netherlands) equipped with eight source measurement units was used for the electrical characterization of the device during exposure. A 50 mV source-drain bias was applied and *V*_GS_ was applied such that the device is operated in depletion mode in the linear regime (*V*_SD_ ≪ *V*_GS_). The drain current (*I*_D_) was measured while the gate potential (*V*_GS_) was swept. This can be applied either via the back gate or the liquid gate. From these characteristics the threshold voltage (*V*_T_) was determined.

To study the influence of the liquid medium in contact with the SiNW on the device characteristics 1,4-dioxane (anhydrous, 99.8%, Sigma-Aldrich Chemie B.V., Zwijndrecht, The Netherlands) (*ε*_r_ = 2.25) and de-ionized water (*ε*_r_ = 80.1; *ρ* = ∼20 kΩ cm) were used as solvent because they mix in all ratios and make it possible to change the dielectric constant gradually in the range of 2–80. They were mixed as described by Åkerlöf *et al.* [37] to obtain mixtures with a range of dielectric constants. To adjust the electrical conductivity, tetramethylammonium chloride (≥98%, Sigma-Aldrich Chemie B.V., Zwijndrecht, The Netherlands) was dissolved in the solvent mixtures where mentioned. The conductivity and pH of the solutions were measured using a Metrohm 712 Conductometer and a Metrohm 827 pH lab meter, respectively (Metrohm equipment was purchased from Applikon Analytical B.V., Schiedam, The Netherlands).

## 3. Results and Discussion

First the devices were exposed to water and the electrical characteristics were determined using the back gate and the liquid gate. The results of this comparison are discussed in Section 3.1. Subsequently, the devices were exposed to water–dioxane mixtures with a range of dielectric constants and the electrical characteristics were determined using liquid gating via an Ag/AgCl electrode (Section 3.2). In addition, the conductivity of some mixtures was adjusted to obtain solutions with similar conductivities.

### 3.1. Back Gate vs. Liquid Gate in De-Ionized Water

**Figure 2.**
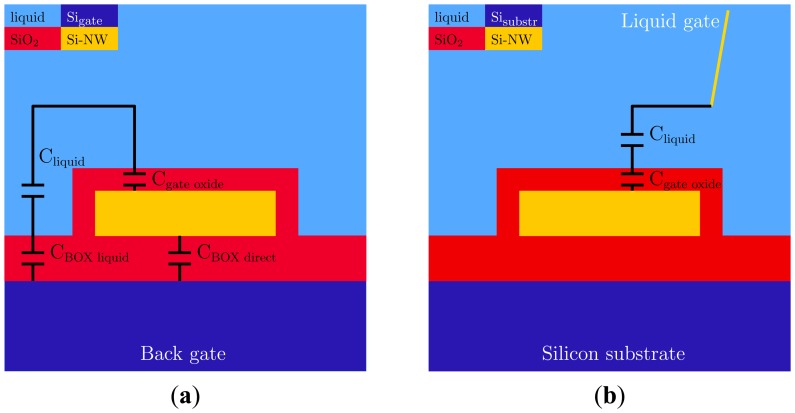
Schematic representations (not to scale) of the coupling of the potential when applying the potential to (**a**) the back gate and (**b**) the liquid gate.

**Figure 3.**
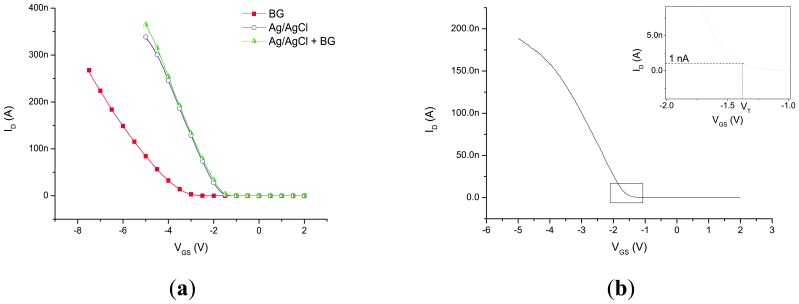
(**a**) *I*_D_-*V*_GS_ characteristics of a device when exposed to water, showing three different types of modes of applying a gate potential. The gate potential was either applied via the back gate without using the liquid gate (red squares), via an Ag/AgCl electrode/liquid gate (blue circles) with the back gate at ground, or via both the back gate and liquid gate via an Ag/AgCl electrode (green triangles); (**b**) Example of the analysis of the curves in de-ionized water (here: liquid-gate swept and back-gate grounded): the threshold voltage (*V*_T_) is the potential at which the current threshold of 1 nA is crossed.

Figure 2 shows a schematic representation of the back-gated and liquid-gated situation and the capacitances that are present. In both cases the *C*_liquid_ was present, although it has a different value for the two cases, while *C*_box_ only plays a role in the case of back gating. The *I*_D_-*V*_GS_ characteristics that were obtained using three different types of modes of operation are given in Figure 3a: (1) the back gate was swept while no electrode is inserted in the solution; (2) the gate potential was applied via the Ag/AgCl electrode while the back gate is connected to ground; and (3) the gate potential was applied to the back gate and liquid gate simultaneously. In more detail, the figure shows the comparison between the use of the back gate and the liquid gate when the device is exposed to de-ionized water (red squares *vs.* blue circles). As expected for p-type nanowires in depletion mode, a more negative gate bias leads to an increase in the drain current, for both types of gating. It is clearly visible that a smaller potential on the liquid gate has to be used compared to the back-gate mode of operation in order to obtain a similar drain current through the nanowire. The addition of the BG to the LG did not have much influence on the characteristics of the device compared to the LG only (blue circles *vs.* green triangles).

To quantify this difference, the total capacitance of the system was estimated for both back and liquid gating as schematically shown in Figure 2, excluding *C*_liq_. Exclusion is justified because this capacitance is not dominating the system, as the electrical conductivity of the de-ionized water is ∼15–30 µS/cm and thus much higher than the conductivity of the SiO_2_ layers (∼10^−16^ S/cm [38]). After calculation of the total capacitances, the threshold voltage can be determined using the estimated total capacitance and the MOSFET (metal oxide semiconductor field-effect transistor) formula for *V*_T_ [39]:

(1) $$V_{\text{T}}=2\varphi_{B}-\frac{\sqrt{2\varepsilon_{\text{S}}qN_{\text{A}}(2\varphi_{\text{B}})}}{C_{\text{total}}}$$

where *φ*_B_ is the difference of the Fermi levels of doped and intrinsic silicon, *ε*_S_ the relative permittivity of the silicon, *N*_A_ the acceptor density, *q* the elementary charge (1.6 × 10^−19^ C), and *C*_total_ the capacitance of the combined dielectric layers. Using estimated values of the total capacitance of 2.0 × 10^−16^ F and 3.1 × 10^−15^ F for the back-gate and front-gate situations, respectively, threshold voltages of −2.8 V and −0.8 V for BG and LG, respectively were calculated. In the experiments a difference of approximately 2 times was found between the BG and LG. The calculated *V*_T_ of the BG configuration is about 3.5 times larger than the one of the LG configuration. This discrepancy can be related to the parallel-plate assumption that was made in the calculation, while the system under study consists of a more complicated geometry. Different densities of trapped charges in the two oxide layers (*i.e.*, BOX and gate oxide) may also contribute to the observed difference.

It is noted that an LG can only be used when there is a continuous electrical path between the Ag/AgCl electrode and the NW-FET via the liquid, as is the present case. Thus, when studying gas or vapour environments one is forced to use the BG or performing differential measurements, while in the case of a liquid contact one can choose. As the BG is not directly exposed to the solvent, its potential will not be influenced by interactions with this solvent. This can be a reason to prefer BG over LG. Furthermore, in terms of fabrication, it is cheaper and easier to use the BG, since no extra processing is needed, while integration of an on-chip electrode does. However, because of the smaller potentials that can be used and the concomitant longer device life times we observed, it was decided to use the LG in the following experiments.

### 3.2. Water–Dioxane Mixtures

**Figure 4.**
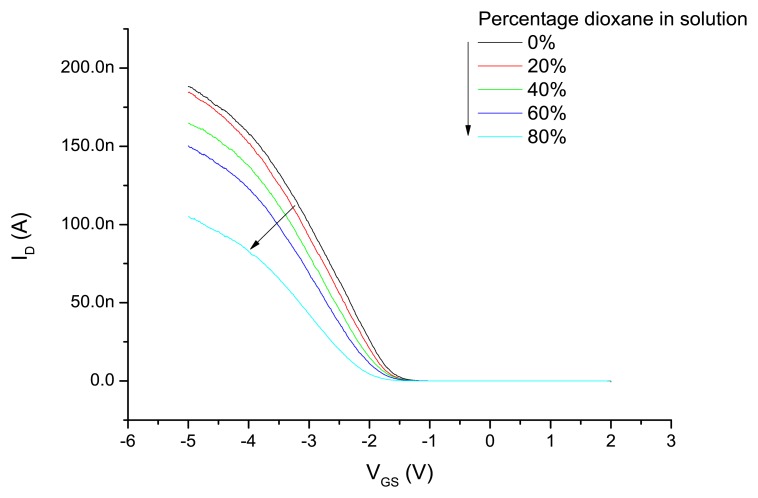
*I*_D_-*V*_GS_ characteristics of a single nanowire device when exposed to the different water–dioxane mixtures. An Ag/AgCl electrode was used as liquid gate in this experiment.

**Figure 5.**
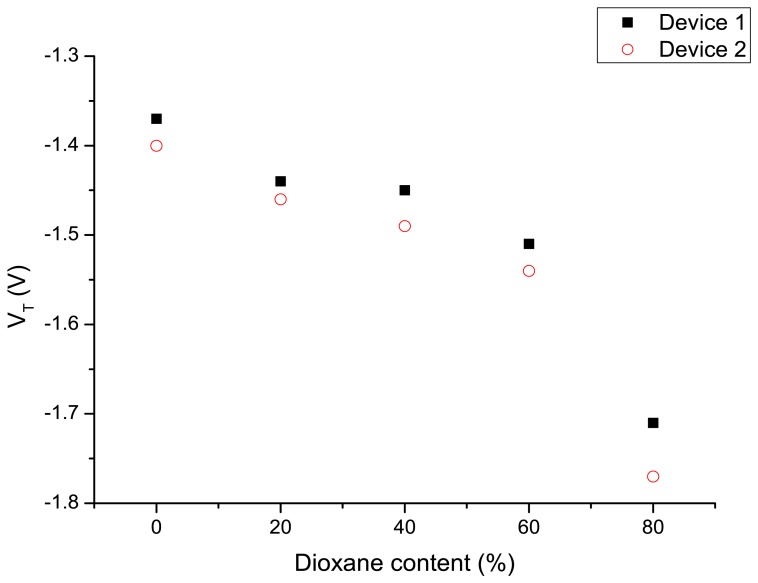
Average threshold voltage of two typical devices as a function of the dioxane content in the water–dioxane mixture exposed to the device.

Subsequent to the experiments with the different modes of applying the gate potential, the device was exposed to water–dioxane mixtures and the *I*_D_-*V*_GS_ characteristics are measured as described above by applying a variable potential to the Ag/AgCl electrode and the back gate connected to ground. The results are shown in Figure 4. Increasing dioxane content of the water–dioxane mixture required the application of a more negative gate potential to arrive at the same drain current. From the *I*_D_-*V*_GS_ curves the threshold voltages were determined and plotted as a function of the dioxane content in Figure 5. A more negative *V*_T_ was observed with increasing dioxane content.

The origin of this observation can be attributed to a decrease of capacitance of the liquid with increasing dioxane content, resulting in an increased negative gate potential to keep the source-drain current constant. However, it is also possible that the reduced dielectric constant of the liquid reduces the dissociation of the surface silanol groups at the oxide interface. This results in a decreased surface charge at a certain proton concentration in the solution. In that case an apparent lower pH value is measured. To compensate for this reduced negative surface potential a more negative potential has to be applied on the gate electrode. To estimate the effect we compare the p*K*_a_ values of protonated water and protonated dioxane, which are −1.74 and −2.92, respectively [40]. Therefore, protonated dioxane is slightly more acidic than protonated water, and concordially water is a stronger base than dioxane.

It is thus expected that upon increasing the content of dioxane in the water–dioxane mixtures, proton dissociation of the surface silanol groups is reduced. This is in line with an experimental study on the acid dissociation constants of several acids in water–dioxane mixtures [41]. We measured the pH of water–dioxane mixtures using a glass electrode and found that the pH meter reading decreased upon the addition of dioxane. This decrease can also be explained by the decreased dissociation of silanol groups now at the glass electrode surface [42]. In addition, the ionic product of water (p*K*_w_) will also decrease upon increasing dioxane fraction [43]. However, that effect reduces the proton concentration, and—if dominant—will increase the surface silanol group dissociation. Since that was not observed in the nanowire experiments, it is concluded that this effect is not dominant. Returning now to our observations on the SiNW-based devices, both the reduced dielectric constant of the water–dioxane medium, *i.e.*, a lower capacitance of that medium, and the reduced dissociation of the surface silanol groups explain the increase *V*_T_ necessary to restore the nanowire conductance.

**Figure 6.**
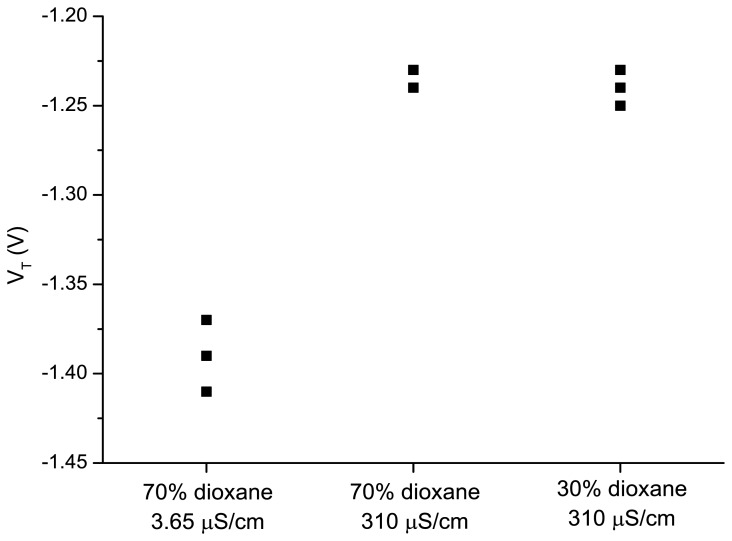
The threshold voltages of the device when exposed to three different water–dioxane mixtures.

To discriminate between these two possibilities we have investigated the change of the medium capacity, by changing the electrolyte concentration and therefore the electrical conductance. A higher fraction of dioxane decreases the conductance and decreases the capacity. A less negative *V*_T_ is thus expected for a higher capacity. We have performed the experiments with the following solutions: (a) water/dioxane (30:70 v/v) with a conductivity of 3.65 µS/cm; (b) water/dioxane (30:70 v/v) with tetramethylammonium chloride added to obtain a conductivity of 310 µS/cm; and (c) water/dioxane ratio (70:30 v/v) with tetramethylammonium chloride added to obtain a conductivity of 310 µS/cm. It was observed (Figure 6) that changing the dioxane/water ratio from 30/70 to 70/30 (v/v) at a constant conductivity of 310 µS/cm did not influence the threshold voltage. Reduction of the conductivity to 3.65 µS/cm for a water/dioxane ratio 30/70 (v/v), resulted in a decrease of the threshold voltage. This shows that the conductivity has a larger effect on the gate potential to be applied for maintaining the drain current than changing only the ratio of water to dioxane.

Therefore we come to the conclusion that the effect of water–dioxane ratios on the electrical properties of SiNW are induced by differences in the capacities of the medium between the nanowire and fate electrode as well as by a reduced dissociation of the surface silanol groups.

## 4. Conclusions and Outlook

In this study, it was demonstrated that there is a different coupling of the gate potential to the SiNW-based device when this potential is applied via the back gate compared to application via a liquid gate. This difference is explained by the BOX layer through which the potential is coupled in the case of the back-gate configuration. This capacitance plays a dominant role over the other relevant capacitances involved. The capacitance of the solution is much less relevant because of its conductivity. Consequently, a reasonable approximation of the system is obtained, even when this layer is not taken into account in the capacitance of the system. Local addition of an individual back gate, which only acts through the area right below the SiNW and which has no capacitive coupling through the liquid, could make a quantitative description of the BG easier. It would also make it possible to individually gate the devices, providing more possibilities for sensor arrays with individual properties.

Furthermore, it was shown that the device characteristics, and most importantly the threshold voltage, are influenced by the solution to which the device is exposed. It is concluded that the change in the threshold voltage as function of dioxane fraction in the medium is determined by a decreased dissociation of the surface silanol groups and a reduced conductivity. Studying the dielectric coupling through the solution will thus only be possible when (1) the surface is passivated with a modification layer which has no interaction with the used solutions and (2) the solutions have a constant conductivity.

When the complete characteristics are taken into account (*V*_T_ and the slope of the *I*_D_-*V*_GS_-characteristics, *i.e.*, the transconductance) an even more complete picture of the device characteristics and the influence of certain solvents could be obtained. Combined with assisted computer learning as demonstrated previously by Niskanen *et al.* [32] a sensor can be constructed from this device. A more detailed approach is needed to explain the observed effects quantitatively, which is beyond the scope of this article. This approach should include a better definition of the capacitance of the solution layer, the charges at the solvent-oxide interface should be taken into account, and adjustments for the absence of a pn-junction need to be added as well as including the presence of mobile charges in the solvent. However, with the simple approach presented in this study it is demonstrated that it is possible to distinguish the different solutions, depending on their conductivity.

To summarize, the results in this article: (1) lead to a better understanding of the electrical response of SiNW-based devices for BG *vs.* LG modes of operation; and (2) show that working in constant ionic strength is required in aqueous solutions and that corrections for the conductivity of mixed-media solutions are essential.
